# Perceptions of national wealth and skill influence pay expectations: replicating global hierarchy on a microscale

**DOI:** 10.3389/fpsyg.2015.00703

**Published:** 2015-05-27

**Authors:** Angela T. Maitner, Jamie DeCoster

**Affiliations:** ^1^Department of International Studies, American University of SharjahSharjah, UAE; ^2^Center for Advanced Study of Teaching and Learning, University of Virginia, CharlottesvilleVA, USA

**Keywords:** status, inequality, system justification, national wealth, stereotypes

## Abstract

In highly multicultural societies, the economic status hierarchy may come to mimic the hierarchy of global wealth, reinforcing social inequality by tying pay scales to national wealth. We investigated how nationality influences *expectations* of payment in the UAE. Participants reported how much they expected people to be paid and how much skill they were perceived to have by nationality. They also reported their perceptions of the national wealth of different countries. Participants generally expected Westerners to be paid more than Arabs, who would be paid more than Sub-Saharan Africans and Asians. Expectations about payment in private sector employment were driven by both actual and stereotyped differences in national wealth and skill, with non-Gulf Cooperation Council Arabs most likely to see national wealth as a factor explaining the economic hierarchy. These results suggest that people expect payment to be tied to national wealth, reflecting the global hierarchy on a microscale.

## Introduction

Disparities in national wealth are striking. Qatar, for example, produces a gross domestic product (GDP) per capita of $102,100 while the Democratic Republic of the Congo has a GDP per capita of only $400 (The World Factbook, 2015). Such disparities have been tied to explanations ranging from differences in national intelligence (i.e., blaming the poor; Lynn and Vanhanen, 2006) to a history of colonization and neoliberal economic policies (i.e., blaming the rich; Hickel, 2013). The current work looks at how the simple *existence* of national wealth disparities, regardless of their cause, may help maintain global inequality in a single context.

Research on system justification theory (see Jost and Banaji, 1994) argues that justifying and explaining status differences helps maintain stability within a system by promoting norms favoring dominant groups. This paper investigates how existing global inequality may be used to understand and explain status differences in a highly diverse society, focusing on how both perceptions of and actual differences in national wealth may be used to understand a nationality-based economic status hierarchy at a local level. We also investigate how perceived and actual merit, which has more commonly been examined as an ideological explanation for the position of disadvantaged group members (see Jost and Hunyady, 2005), influence individuals’ expectations of salary differences.

System Justification Theory argues that people are motivated to perceive the norms, rules, and structures within their societies as legitimate, even when those structures disadvantage them as individuals. In fact, Jost et al. (2003) argue that the motive to justify unequal status in society should be particularly strong for members of disadvantaged groups, who experience the most ideological dissonance in trying to understand their low status in society.

A number of other factors influence the system justification motive as well. Kay and Friesen (2011) argue that people are motivated to defend the social order when they are dependent on the system, when they perceive a lack of personal control, when the system is under threat, or when the system is inescapable (see also Laurin et al., 2010). Likewise, system longevity (Blanchar and Eidelman, 2013), a sense of powerlessness (van der Toorn et al., 2015), or individual differences in need for order, structure, and closure, openness to experience, and perceptions of the world as a dangerous place (Jost and Hunyady, 2005) increase defense of the status quo.

Defense of the status quo takes several shapes, one of which is the creation and maintenance of stereotypes that explain the relative status of groups within society. Jost et al. (2005), for example, showed across three national samples that high-status groups tended to be stereotyped as agentic, whereas low status groups were perceived as communal. These stereotypes were observed among both high- and low-status group members and could be used to legitimize an existing social order by supporting the idea that groups with skill achieve (and therefore deserve) higher status in society.

A growing body of research has shown evidence of system justification in regions outside of Western, capitalistic societies, where the system is expected to reward such effort and ability. For example, Henry and Saul (2006) showed that low-status school children in Bolivia believed that the government was effectively meeting people’s needs even more than their high status counterparts, providing evidence of system justification in a developing country. van der Toorn et al. (2010) found evidence of system justification in the U.S. and Hungary, but showed that whereas endorsement of system-justifying ideologies was associated with more favorable views of work scenarios that emphasized equity (merit-based payment) in the U.S., endorsement of system-justifying ideologies in Hungary was associated with more favorable views of scenarios that emphasized equality and the favoring of a coworker over the self (a communist value). In a review of system justification in capitalist and post-Communist societies, Cichocka and Jost (2014) argued that although there is a weaker degree of system justification in post-Communist societies overall, system justification has similar antecedents, manifestations, and consequences across all sampled societies. Taken together, these studies suggest that system justification is a global phenomenon, although the extent to which it is endorsed and the specific status-oriented scenarios that are favored may vary with local systems and norms.

The current work investigates how status differences are understood in the UAE, a modern emerging economy comprising a globally diverse workforce. The system in the UAE is stable with laws that reduce the possibility and probability of collective mobility (see Issa, 2006). According to previous research, this lack of personal control is likely to motivate strong support for the status quo, or at least demand explanations for social hierarchy (i.e., Laurin et al., 2010; Kay and Friesen, 2011), especially from individuals who occupy low status positions (Jost et al., 2003).

The economic system in the UAE is highly stratified. Salary surveys show that, among individuals in the private sector working at the same job, Western expatriates tend to make more than Arab expatriates, who tend to make more than South Asian expatriates (Al-Awad and Elhiraika, 2003; Nagraj, 2013; Pant, 2013), although the gap between Western and Arab expatriates has recently decreased (Anderson, 2014). Such salary differences are part of the national dialog (Salama, 2004; Al Subaihi, 2012), and are well known to residents and research participants, many of whom seem to expect merit to play a role alongside nationality in salary decisions (see Maitner, 2015).

There are several possible ways that individuals may understand the nationality-based hierarchy. First, because there are national differences in education, it may be that some nationalities are actually better qualified and prepared for work than others, and are consequently paid more for their superior skills. An alternative possibility is that, like in Western countries, higher status groups may be *perceived* as more qualified than lower status groups, independent of any true differences in competence. If this is the case, then *stereotypes* of national level qualifications may influence how much individuals of different nationalities earn.

Alternatively, the economic status hierarchy may be unrelated to national differences in either perceived or actual qualifications, and may instead simply reflect the global economic hierarchy, either in actuality, or in how it is perceived. Arab culture tends to be hierarchical such that people who have more *wasta*, or social influence based on individual connections, are expected to achieve better outcomes than those with less social influence (see Cunningham and Sarayrah, 1994). In other words, in the local system, individuals expect ascribed status to strongly influence people’s outcomes. Following van der Toorn et al. (2010), we may therefore expect such local values to play a role in the way individuals justify or explain the current system. If this is the case, it may be that ascribed status associated with one’s nationality will be perceived as an especially potent explanation for socioeconomic status within the UAE. Additionally, given that 89% of residents (in 2010) are foreigners (United Arab Emirates National Bureau of Statistics, 2011), ascribed status associated with the economic power of one’s nationality may serve as a simple and direct explanation for salary discrepancies. In this case, actual or perceived differences in national wealth should play a role in how much individuals expect people from different nationalities to earn.

According to *Gulf Business*, “Local companies are still willing to pay a premium for Western expatriates from outside the region, due to the perception that they possess a particular skill set not locally available and that they have to be paid comparable rates for the same position in their home country….” (Nagraj, 2013). In other words, business experts assert that stereotypes of *both* skill differences and national economies play a role in explaining salary hierarchies. Such stereotypes may help institutionalize and perpetuate a system of global inequality on a microscale.

Wakslak et al. (2011) have argued that once a system justification motive has been activated via system threat, individuals may aim to justify the system at different levels, from microscale systems such as families and friendship cliques to larger-scale systems such as national economies. Their results suggest that individuals may defend multiple social systems even when only one system has been threatened, an effect they call spreading rationalization. Here we investigate whether the hierarchy of a superordinate system may be used to explain the hierarchy in a subordinate system that individuals are motivated to defend. In this way, both systems may be legitimized and inequality perpetuated.

This study investigates the contribution of actual nation characteristics (i.e., GDP per capita and mean education levels – reflecting the actual global hierarchy) and national stereotypes (i.e., perceived wealth and skill – reflecting the stereotyped global hierarchy) on expected salaries for employees with different nationalities. Therefore it is also pitting markers of ascribed status, either real or perceived (GDP per capita and perceived wealth), against markers of earned status (mean education levels and perceived skill) to investigate how the two kinds of explanations are used to understand socioeconomic hierarchy in a non-Western economy. Using a diverse sample of UAE-based students, we first examine whether stereotypes about the agency (merit) and success (wealth) of different groups reflect the relative status they hold in UAE society, expecting nationalities perceived as higher on the economic status hierarchy (nationalities expected to be paid higher salaries) to be perceived as more agentic and successful (see Jost et al., 2005). Second we investigate the role that structural variables and stereotypes play in explaining the perceived economic hierarchy, expecting stereotypes about national merit and wealth to be used as explanations for perceived nationality-based payment differences. Third, we investigate whether the extent to which individuals use wealth or skill-based explanations for economic differences varies by participant nationality, expecting participants lower in the economic hierarchy to show stronger motives to explain status differences (see Jost et al., 2003). Finally, we examine whether the extent to which individuals use wealth or skill-based explanations for economic differences varies by target nationality; here we explore whether people use different explanations for salaries of groups who hold different positions in society or whether the same explanations are used across all groups, regardless of their relative status.

## Materials and Methods

### Participants

One hundred ninety-five students (*M* age = 19.98, SD = 1.60; 78 male, 116 female, 1 unreported; 1 American, 2 Bangladeshi, 2 British, 2 Canadian, 1 Chinese, 19 Egyptian, 39 Emirati, 1 Filipino, 1 German, 1 Hungarian, 22 Indian, 3 Iranian, 3 Iraqi, 13 Jordanian, 1 Kuwaiti, 5 Lebanese, 1 Mozambican, 5 Nigerian, 16 Pakistani, 11 Palestinian, 7 Saudi, 1 Somali, 1 Sri Lankan, 5 Sudanese, 10 Syrian, 1 Tajik, 1 Tanzanian, 1 Turkish, 1 Ukrainian, 8 multiple nationalities, 10 unreported) from the American University of Sharjah completed the survey for partial course credit. Although the restricted sample is not representative of the country as a whole, university students represent a group who will soon enter the workforce with strong educational credentials. This sample therefore represents a nationally diverse, skilled group for whom the issue of salary is personally relevant. Moreover, because they have not yet entered the workforce, participants’ perceptions are likely to be reflective of local expectations rather than personal experience, allowing us to investigate the perceptions they report as stereotypes.

### Procedure

The following procedure was approved by the Institutional Review Board at the American University of Sharjah.

Participants were told that the study investigated the impact of nationality on pay and ability. All participants began by reading and signing informed consent documentation. They then received all instructions and completed the study on computers running MediaLab software (Jarvis, 2012). They were asked to provide their first responses to the questions that followed, reporting expected salary and perceptions of qualifications and national wealth for 29 different nationalities which are visibly present in the UAE.

These 29 independent nationalities were categorized based on region for analysis purposes. Categories were created in an attempt to identify regional groups that play meaningfully different roles in UAE society and that reside in different positions in the economic hierarchy. Because salary surveys frequently report salary for Western, South Asian, and Arab expatriates, we began with these three groups. However, because member countries of the Gulf Cooperation Council (GCC), comprising Bahrain, Kuwait, Oman, Saudi Arabia, Qatar, and the UAE, have stronger national economies associated with higher levels of ascribed status, we differentiated GCC Arab and non-GCC Arab target groups. Finally, in an attempt to explore perceptions of additional regions, we included Sub-Saharan African and Southeast Asian countries. Target regions included:

(1) Western: American, Australian, British, and French
(2) South Asian: Bangladeshi, Indian, Pakistani, and Sri Lankan
(3) Non-GCC Arab: Egyptian, Moroccan, Sudanese, Tunisian, Jordanian, Lebanese, Palestinian, Syrian, Iraqi, and Yemeni
(4) GCC Arab: Emirati, Kuwaiti, Omani, Qatari, and Saudi
(5) Sub-Saharan African: Ethiopian, Kenyan, Nigerian, and Somali
(6) Southeast Asian: Filipino, Indonesian, and Malaysian

We attempted to use the same breakdown in creating categories of participants’ nationalities, but only had sufficient representation for the South Asian, non-GCC Arab, and GCC Arab regions. All other nationalities were collapsed into a single “Other” region (see **Table 1**).

**Table 1:** Descriptive statistics for study variables.

Level of aggregation	Variable	Mean (SD) or *N* (%)
Rating	Expected Pay	2.79 (1.24)
	Perceptions of national wealth	2.68 (1.28)
	Perceptions of national skill	2.90 (1.15)
Person	Region for participant’s nationality	
	South Asian	43 (22.1%)
	Non-GCC Arab	73 (37.4%)
	GCC Arab	48 (24.6%)
	Other	31 (15.9%)
Nation	Actual GDP per capita	$14,589 ($15,898)
	Actual amount of schooling	12.48 (3.49)
	Region for rated nation	
	Western	4 (14.3%)
	South Asian	4 (14.3%)
	Non-GCC Arab	9 (32.1%)
	GCC Arab	4 (14.3%)
	Sub-Saharan African	4 (14.3%)
	Southeast Asian	3 (10.7%)

#### Expected Salary

Participants began by reporting expected starting salaries by nationality. They were asked to “please imagine that the person described was qualified for a job that had a starting salary range. We will ask you to estimate, given the possible range, how much the person would be likely to be paid.” Participants then responded to the prompt “In the private sector, if a(n) [nationality] was hired for a job for which (s)he was qualified, (s)he would likely be offered a starting salary which was at the _____ of the salary range” using a scale anchored at 1 = *lowest end* and 5 = *highest end*. Participants evaluated the 29 nationalities in a random order.

#### Expected Qualifications

After rating salary expectations for all nationalities, participants were told “for the next several questions, please think about how most people perceive the skill level (a combination of education, ability, and work ethic) of different national groups. We will ask you to estimate, given the possible range, how much skill a person with a particular nationality is expected to have.” They then evaluated the 29 nationalities in a random order, on the item: “A(n) [nationality] is expected to have skills (a combination of education, ability, and work ethic) at the __________ of a scale compared to other nationalities,” using a scale anchored at 1 = *lowest end* and 5 = *highest end*.

#### Expected National Wealth

Finally, participants were asked to rate the national wealth of the 29 countries compared to other countries. Here they responded to the item “The national wealth of [country] is at the ___________ compared to other countries,” again using a scale anchored at 1 = *lowest end* and 5 = *highest end*. Participants then reported personal demographic information before being thanked and debriefed.

#### National Statistics

After data were collected from participants, we retrieved the most recent available data on GDP per capita (the total value of goods and services produced by a nation valued at prices prevailing in the U.S., then divided by the total population –taken as an indicator of national wealth) and school life expectancy (average number of years of schooling – taken as an indicator of national qualifications) from the World Factbook, a data repository maintained by the U.S. Central Intelligence Agency that provides a diverse collection of information on 267 world entities.

## Results

### Outliers

Before performing our main analyses, we graphically examined the distributions of our main variables to identify potential outliers. In our investigation of the nations being rated, we noted that that GDP per capita for Qatar ($102,100) was almost twice as large as the second highest GDP per capita ($52,800) and 3.75 SD greater than the mean GDP per capita ($17,606). We therefore decided to remove the ratings of Qatar from our analyses so that they would not bias results. We did not have any participants from Qatar, so we did not need to investigate the possibility of Qatari participants being outliers on any ratings.

### Descriptive Statistics

Descriptive statistics for the variables included in our analyses appear in **Table 1**.

The data for the present study was collected at three different levels. The 195 participants evaluated the pay, wealth and skill of the 28 target nationalities (excluding Qatari) for a total of 5460 ratings. Each of the 195 participants also reported their own nationality, which was then used to determine participant region. Finally, information about the GDP per capita and average amount of schooling were retrieved for the 28 target nationalities. The descriptive statistics for each of our variables was calculated based on the level at which the data were collected. Therefore, the statistics for rated pay, wealth, and skill were calculated based on the 5460 ratings, the counts for participant region were calculated based on the 195 reports from participants, and the statistics for actual GDP and amount of schooling were calculated based on the 28 reports from the rated nations.

Given that our variables were collected at different levels of aggregation, we decided to examine the bivariate relations among variables at multiple levels of aggregation. **Table 2** presents the correlations among the perceived and actual nation characteristics.

**Table 2:** Correlations among rated and actual nation characteristics by level of aggregation.

	1	2	3	4	5
**Rating level**
1. Perceptions of pay	-				
2. Perceptions of wealth	0.67	-			
3. Perceptions of skill	0.47	0.46	-		
4. GDP per capita	0.64	0.71	0.39	-	
5. Average amount of schooling	0.52	0.58	0.35	0.78	-
**Nation level**
1. Perceptions of pay	-				
2. Perceptions of wealth	0.95	-			
3. Perceptions of skill	0.83	0.77	-		
4. GDP per capita	0.90	0.93	0.78	-	
5. Average amount of schooling	0.74	0.78	0.69	0.78	-

All correlations are significant (*p* < 0.005).

Since national stereotypes were assessed at the rating level and the actual nation characteristics were assessed at the nation level, we present the correlations at both of these levels of aggregation. All of the correlations are positive, strong, and significant. The correlations at the nation level are expectedly higher because the nation-level aggregates of perceived characteristics average over person-level variability (which is included in the individual ratings). As can be seen, perceptions of wealth and actual wealth were highly related, as were perceptions of skill and amount of schooling, indicating that participants’ stereotypes were largely, but not wholly, accurate.

Participant nationality was assessed at the person level, so we used a data set aggregated to the person level to test the bivariate relations of the participant’s region with expected pay, perceived wealth, and perceived skill. A one-way between-subjects ANOVA indicated that participant region was significantly related to perceived wealth [*F*(3,191) = 3.65, *p* = 0.01], such that those from GCC nations provided significantly lower ratings of wealth (*M* = 2.54, SD = 0.45) than those from other nations (*M* = 2.73, pooled SD = 0.37). One-way between-subjects ANOVAs indicated that participant region was not significantly related to either expected pay [*F*(3,191) = 1.73, *p* = 0.16] or perceived skill [*F*(3,190) = 0.50, *p* = 0.68]. This suggests that at an overall level, there is remarkable consistency across the diverse participant pool in ratings of national wealth, pay, and skill, with the exception of GCC Arabs rating national wealth lower across national groups. Overall, different groups living in the UAE share stereotypes about different nationalities.

### Stereotypes and the National Hierarchy

To test our first research question asking whether stereotypes about the merit and wealth of different groups reflect the relative status they hold, we examined the bivariate relations of region being rated with perceived nation characteristics. To secondarily assess how closely those stereotypes reflect global reality, we also assessed the bivariate relations of region being rated with actual nation characteristics. Region of the nation being rated was assessed at the nation level, so we used a data set aggregated to the nation level to investigate these relations. The means of and homogenous subsets for these variables by region are provided in **Table 3**.

**Table 3:** Means and homogenous subsets of perceived and actual nation characteristic by rated region.

Rated region	Expected pay	Perceived wealth	Perceived skill	GDP per capita	Average schooling
Western	4.23_D_	4.22_C_	4.13_D_	$42,200_C_	17.25_C_
South Asian	1.91_A_	1.93_AB_	2.59_AB_	$3,925_A_	11.00_AB_
Non-GCC Arab	2.69_B_	2.30_B_	2.87_BC_	$6,333_A_	11.25_A_
GCC Arab	3.82_C_	3.98_C_	2.90_C_	$33,275_B_	15.00_BC_
Sub-Saharan African	1.96_A_	1.76_A_	2.30_A_	$1,625_A_	9.00_A_
Southeast Asian	2.06_A_	2.29_AB_	2.63_AB_	$9,133_A_	12.33_AB_

Means within a column that share a subscript are not significantly different from each other (LSD *p* > 0.05).

A collection of one-sample between-subjects ANOVAs indicate that the rated region had significant effects on all of these variables (all *p*’s < 0.005). Typically, Western nations had the highest ratings, followed by GCC Arab nations, followed by the other nations. The effects were strongest for expected pay and GDP per capita (*R*^2^s > 0.90) and weakest for the average amount of schooling (*R*^2^ = 0.57).

As can be seen in **Table 3**, participants expected individuals from Western nations to be paid more than individuals from GCC Arab nations, followed by non-GCC Arab nationals. Participants expected individuals from South Asian, Sub-Saharan African, and Southeast Asian nations to earn the least for the same job. These findings denote the perceived economic hierarchy in the UAE, and show that participants have a fair idea of how much individuals with different nationalities are actually paid in a relative sense (see Nagraj, 2013; Pant, 2013).

Participants also perceived nationals from Western states to be more skilled than nationals from GCC Arab states, followed by nationals from non-GCC Arab states, then South and Southeast Asian states, and finally Sub-Saharan African states. In other words, stereotypes about the agency of different segments of society roughly mirror the expected payment those members of society are expected to receive. This finding reflects that of previous research (Jost et al., 2005) with individuals from nations expected to be higher in the economic hierarchy also expected to possess more skill.

Stereotypes about national wealth also roughly mirror the expected payment hierarchy. The more payment individuals from a region are expected to receive, the more wealth that region is expected to have. Although actual national wealth (GDP per capita) also mirrors somewhat the hierarchy seen in expectations of payment by region, it fails to differentiate between non-GCC Arab, South and South East Asian, and Sub-Saharan African countries. This suggests that the perceived status hierarchy in the UAE (the hierarchy reflecting expected pay) is more nuanced and differentiated than global economic reality.

Bivariate correlations reported in **Table 2** provide further support for the idea that stereotypes about the merit and wealth of different groups reflect the relative status they hold, showing that perceptions of payment are highly associated with perceptions of and actual differences in national wealth and skill.

### Predicting Expected Pay from Stereotypes and National Characteristics

To examine our second research question investigating how both stereotypes of and actual estimates of national wealth and skill relate to expected pay, we ran a linear mixed model in SPSS (version 21) predicting expected pay from stereotypes of national wealth, stereotypes of national skill, actual national GDP per capita, and actual national years of schooling on data at the rating level while adjusting for participant as a cluster variable. The continuous predictor variables were all group-mean centered (although this is the same as grand mean centering for actual GDP per capita and average years of schooling since these values did not vary between participants). After centering, the predictors were all divided by their standard deviation so that coefficients from the model represent expected change in the outcome with a 1 standard deviation change in the corresponding predictor. The model allowed for random intercepts and random slopes for the ratings of national wealth and national skill because the meaning of these two variables could vary between participants. All of these random effects were significant (all *p*’s < 0.05), indicating that the mean ratings of expected pay and the relations of ratings of wealth and skill with the rating of expected pay varied significantly across participants.

We also included the region of the nation being rated and the region of the participant’s nationality in the model as categorical blocking factors. In analyses, the rated region was represented by a set of dummy codes with “Southeast Asian” being the reference group. The participant’s region was represented by a set of dummy codes with “Other” as the reference group.

Omnibus tests indicated that the overall test for rated region was significant [*F*(5,4055) = 120.54, *p* < 0.001], but the overall test for participant’s region was not significant [*F*(5,170) = 1.20, *p* = 0.31]. Means and homogeneous subsets for rated region are presented in **Table 4**.

**Table 4:** Means and homogenous subsets for the main effect of rated region.

Rated region	Mean expected pay
Western	3.360_E_
South Asian	2.295_A_
Non-GCC Arab	2.864_C_
GCC Arab	3.059_D_
Sub-Saharan African	2.481_B_
Southeast Asian	2.289_A_

Means not sharing the same subscripts are significantly different from each other (*p* < 0.05).

These means differ somewhat from those presented in **Table 3** because they control for the effects of perceived and actual nation characteristics, as well as the effect of participant region. As expected, and reflecting the bivariate analyses reported above, the highest pay was expected for individuals from Western nations, whereas the lowest pay was expected for individuals from South Asian and Southeast Asian nations.

The coefficients for the fixed effects in this model are presented in **Table 5**.

**Table 5:** Coefficients from model testing main effects on expected pay.

Predictor	Estimate (SE)	*p*-value
Intercept	2.249 (0.094)	<0.001
Perceived national wealth	0.308 (0.026)	<0.001
Perceived national skill	0.189 (0.020)	<0.001
GDP per capita	0.220 (0.049)	<0.001
Average amount of schooling	0.040 (0.015)	0.008
Rated region		
Western dummy	1.071 (0.077)	<0.001
South Asian dummy	0.006 (0.037)	0.88
Non-GCC Arab dummy	0.575 (0.032)	<0.001
Sub-Saharan African dummy	0.192 (0.041)	<0.001
GCC Arab dummy	0.771 (0.065)	<0.001
Participant’s region		
South Asian dummy	0.218 (0.117)	0.06
Non-GCC Arab dummy	0.055 (0.106)	0.61
GCC Arab dummy	0.031 (0.114)	0.79

Continuous predictors are centered and on a standardized metric, so the coefficients represent the expected change in expected pay with a 1 SD increase in the predictor.

These results indicate that expected pay was higher when the participant perceived the nation as being wealthier or having individuals with more skill, and when the nation actually had a higher GDP per capita or higher average amount of schooling. In other words, in line with assessments of business professionals, participants seem to expect salary to follow from both national wealth and qualifications, with stereotypes and actual differences in those variables playing independent roles in influencing participant expectations.

### Moderating Effects of Participant’s Region

To test our third research question, we investigated whether the geographic region a participant was from influenced observed relations between national stereotypes and expected pay. We expected participants from regions lower on the economic status hierarchy (non-GCC Arabs and South Asians) to show a stronger need to explain the hierarchy, and therefore to show stronger relations between stereotypes and expected pay. To test this hypothesis, we ran a linear mixed model that predicted expected pay from the main effects discussed above plus the interactions of the participant’s region with perceived wealth and perceived skill. We decided not to examine the interactions of participant’s region with the actual nation characteristics (GDP per capita and average amount of schooling) because these interactions are strongly affected by which nations were included in the study and how those nations were divided into regions, which were researcher choices. The model adjusted for participant as a cluster variable. Centering was used in the same way as in the prior model. The model allowed for random intercepts and random slopes for the ratings of national wealth and national skill. All of these random effects were significant (all *p*’s < 0.05). Interaction terms were tested by including a set of product terms, multiplying each of the region dummy codes by the corresponding predictor variable.

The coefficients for the fixed effects in this model are presented in **Table 6**.

**Table 6:** Interaction coefficients from model testing the moderating effects of the participant’s region.

Predictor	Estimate (SE)	*p*-value
Participant’s region × perceived wealth		
South Asian dummy × perceived wealth	-0.036 (0.076)	0.64
Non-GCC Arab dummy × perceived wealth	0.129 (0.069)	0.06
GCC Arab dummy × perceived wealth	-0.150 (0.074)	0.04
Participant’s region × perceived skill		
South Asian dummy × perceived skill	0.011 (0.066)	0.87
Non-GCC Arab dummy × perceived skill	0.045 (0.060)	0.45
GCC Arab dummy × perceived skill	0.044 (0.065)	0.50

The omnibus test for the interaction between the participant’s region and rated wealth was significant [*F*(3,201) = 7.80, *p* < 0.001], but the omnibus test for the interaction between the participant’s region and rated skill was not [*F*[3,194) = 0.30, *p* = 0.85]. The slopes and homogeneous subsets for rated wealth and rated skill by participant region are presented in **Table 7**.

**Table 7:** Slopes and homogenous subsets for perceived wealth and perceived skill by participant’s region.

Participant’s region	Expected slope for perceived wealth	Expected slope for perceived skill
South Asian	0.269_AB_	0.171_A_
Non-GCC Arab	0.434_C_	0.205_A_
GCC Arab	0.155_A_	0.204_A_
Other	0.305_BC_	0.160_A_

Slopes within a column that share a subscript are not significantly different from each other (LSD *p* > 0.05).

As can be seen, the ratings of wealth are least predictive for those from GCC Arab nations and most predictive for those from non-GCC Arab nations. In other words, GCC Arabs expect national wealth to make a smaller difference in how much individuals are paid, whereas non-GCC Arabs expect national wealth to make a larger difference in how much individuals are paid.

These results suggest that non-GCC Arabs, who are relatively disadvantaged in the local hierarchy, expect national wealth to play a more important role in determining how much individuals are paid than other participants. This may reflect a stronger motive to justify or explain the hierarchy in relatively disadvantaged participants. Importantly, the method they employed seems to reflect local values of hierarchy reflecting ascribed status (i.e., payment follows from national wealth), in contrast to Western values of hierarchy reflecting earned status (i.e., payment follows from national skill).

Indeed, neither non-GCC Arabs nor South Asians were more likely than other participants (GCC Arabs and ‘other’) to use stereotypes about skills to understand the payment hierarchy, suggesting that merit, while an important contributor to participants’ payment expectations overall, may not help reduce dissonance as strongly as considerations of wealth (i.e., ascribed status) in UAE-based, relatively disadvantaged participants.

### Moderating Effects of Region Being Rated

To test our fourth research question, we investigated whether the geographic region of the nation being rated influenced the relations of perceived wealth and perceived skill with expected pay. To examine these interactions, we ran a linear mixed model that predicted expected pay from the main effects discussed above plus the interactions of the region being rated with perceived wealth and perceived skill. The model adjusted for participant as a cluster variable. Centering was used in the same way as in the previous models. The model allowed for random intercepts and random slopes for the ratings of national wealth and national skill. All of these random effects were significant (all *p*’s < 0.05). Interaction terms were tested by including a set of product terms, multiplying each of the region dummy codes by the corresponding predictor variable. The interaction terms were all tested simultaneously.

The coefficients for the fixed effects in this model are presented in **Table 8**.

**Table 8:** Interaction coefficients from model testing the moderating effects of the rated region.

Predictor	Estimate (SE)	*p*-value
Rated region × perceived wealth		
Western dummy × perceived wealth	0.017 (0.062)	0.78
South Asian dummy × perceived wealth	-0.007 (0.060)	0.91
Non-GCC Arab dummy × perceived wealth	0.031 (0.053)	0.56
Sub-Saharan African dummy × perceived wealth	0.068 (0.069)	0.32
GCC Arab dummy × perceived wealth	0.154 (0.057)	0.006
Rated region × perceived skill		
Western dummy × perceived skill	-0.001 (0.051)	0.98
South Asian dummy × perceived skill	-0.033 (0.048)	0.50
Non-GCC Arab dummy × perceived skill	0.017 (0.045)	0.70
Sub-Saharan African dummy × perceived skill	0.016 (0.056)	0.78
GCC Arab dummy × perceived skill	-0.159 (0.054)	0.003

The omnibus test for the interaction between region rated and rated wealth [*F*(5,4543) = 2.51, *p* = 0.03] and the omnibus test for the interaction between region rated and rated skill [*F*(5,4577) = 3.38, *p* = 0.005) were significant. The slopes and homogeneous subsets for rated wealth and rated skill by participant region are presented in **Table 9**.

**Table 9:** Slopes and homogenous subsets for perceived wealth and perceived skill by rated region.

Rated region	Expected slope for perceived wealth	Expected slope for perceived skill
Western	0.276_A_	0.214_B_
South Asian	0.252_A_	0.183_B_
Non-GCC Arab	0.290_A_	0.233_B_
GCC Arab	0.414_B_	0.056_A_
Sub-Saharan African	0.326_AB_	0.231_B_
Southeast Asian	0.259_A_	0.216_B_

Slopes within a column that share a subscript are not significantly different from each other (LSD *p* > 0.05).

From this, we can see that perceived wealth has the largest impact on expected pay when the rated nation is from the GCC region, and that this relation is significantly stronger than that found for any of the other regions except that for Sub-Saharan African nations. In other words, participants expected GCC Arabs’ salaries to be influenced by their countries’ wealth more than they expected other individuals’ salaries to be influenced by the wealth of their nation of origin. We can also see that rated skill is not perceived as important when assigning pay to individuals from GCC Arab nations, but is positively related to the rated pay for other regions. In other words, participants did not expect GCC Arabs’ salaries to be influenced by their countries’ expected skill, although this was an important factor for individuals from other nations.

In sum, it seems that the predicted pay for individuals from GCC Arab nations is more strongly influenced by perceptions of national wealth and less strongly influenced by perceptions of national skill relative to other regions.

## Discussion

Results indicated that participants representing different nationalities share a perception of a local economic hierarchy, with Westerners expected to earn more than Arabs, who are expected to earn more than Sub-Saharan Africans and Asians. Moreover, that hierarchy is reflected in stereotypes about national skill and wealth, with nationalities that are expected to be more economically powerful in the local context perceived as more skillful and wealthy. Results further indicated that these stereotypes support differences in expected payment, as both stereotypes about and real differences in national wealth and skill were predictive of salaries for people of different nationalities. A stronger relationship between perceived wealth and payment was found for non-GCC Arabs, who occupy a relatively disadvantaged position in the local hierarchy, though South Asians, those lowest in the local hierarchy, did not seem to rely on either stereotypes about skill or wealth more than other groups to understand payment differences in society. Finally, the results indicated that participants considered national wealth to be a more important contributor and national skill a less important contributor to salaries for GCC Arabs relative to other regional groups.

Our findings suggest that, national stereotypes, including perceptions of wealth and skill, support the status hierarchy, with Western countries perceived as more skilled and wealthy than other regions. These stereotypes appear to have a basis in international reality, with Western nations typically having higher GDPs and amounts of schooling than other regional groups. However, actual GDP and schooling fail to fully explain differences in expected payment, and therefore it is likely that stereotypic perceptions help create and explain a more nuanced local hierarchy, with regions expected to fall lower in the hierarchy *perceived* as less skilled and wealthy than other regions. Like Jost et al. (2005), then, we show the importance of stereotypes in explaining status differences in society. However, we show that in this context, where local norms tie hierarchy to preexisting indicators of influence, explanations tied to ascribed status may be embraced at least as strongly as explanations tied to earned status.

Both actual nation characteristics, including GDP and school life expectancy, and perceptions of national characteristics, including national wealth and skill, independently predicted how much participants expected individuals to be paid. This suggests that participants may be using explanations based in global economic differences to understand the local hierarchy, using the global hierarchy to explain the local one. In this way, individuals may show a different form of spreading rationalization, using knowledge about the macrosystem to explain (and perhaps defend) the microsystem. However, it seems that the local hierarchy is understood as reflective of both privilege and merit, with participants expecting both ascribed status (associated with a nation’s wealth) and earned status (associated with a nation’s skill) to influence salary.

We found that non-GCC Arabs expected national wealth to play a stronger role in determining an individual’s salary than individuals from GCC Arab states or South Asia. Similar to Jost et al. (2003), it appears that individuals lower in the socio-economic hierarchy are more likely to use indicators, in this case national wealth, to understand or predict economic outcomes, reflecting a stronger ideological need to explain the economic hierarchy. Unexpectedly, South Asians, the most disadvantaged group represented in our sample, did not show a similar propensity, and in fact, did not significantly differ from GCC Arabs, the most privileged group within our sample. It may be that individuals holding a local ethnicity have a stronger motivation to explain their position than individuals who do not share as many characteristics with the local population. However, this intriguing difference demands further investigation.

Although perceptions of national wealth predicted salary for individuals from all target regions, it was more predictive of expected salary for individuals from GCC Arab states. This finding may simply reflect knowledge of reality, as UAE nationals earn as much as 44% above an industry standard because of their nationality (Hay Group). It may also reflect the importance of ascribed status in determining individuals’ outcomes, especially in the Arab world. Specifically, the ascribed status associated with national wealth may be expected to strongly influence outcomes for GCC Arab individuals who are nationally economically advantaged. Because non-GCC Arab countries do not typically have high levels of national wealth, they may not be able to rely on such ascribed status to bolster their position.

Consistent with that finding, we also found that perceptions of national skill influenced expected salaries for individuals from all regions except GCC Arabs. Taken together, this suggests that participants expect GCC Arabs to be paid based on national wealth, with qualification and skill playing little or no role in determining salary for individuals from this region. These results add a caveat to the existing literature by highlighting the fact that individuals may not use the same explanations for the status position of all members of society, and instead may rely on different explanations for the position of different groups, following from either realistic or stereotypic perceptions.

In future work, we would like to investigate more directly whether and to what extent explanations based in national wealth and skill are perceived as justified and legitimate explanations for the existing social hierarchy, focusing on the extent to which global wealth disparities may be used to not only explain but legitimize status differences both within and between societies.

We may also investigate whether individuals from lower status nationalities are more satisfied with their position in UAE society when they make comparisons to co-nationals in their country of origin. Research shows that individuals tend to compare their levels of payment to those of ingroup rather than outgroup members and feel satisfied when their payment compares favorably to that of ingroup members, regardless of how much outgroup members are paid (see Bylsma and Major, 1994). If individuals perceive better opportunities in the UAE than at home, they may report satisfaction regardless of their relative position in the UAE status hierarchy. This could help explain why South Asians failed to show a particularly strong reliance on wealth or skill to understand the economic status hierarchy. Non-GCC Arabs, sharing multiple ingroup memberships with GCC-Arabs, may have a stronger propensity to make social comparisons to that group (rather than ingroup members in their home country), and may perceive unfavorable comparisons as a result.

We may also find that non-GCC Arab and South Asian participants, in particular, are more satisfied if their salary compares favorably to that of co-nationals in the UAE, regardless of payment given to other nationals. Such ingroup social comparisons also influence entitlements (Bylsma and Major, 1994), and we may further investigate whether non-GCC Arab or South Asian participants show evidence of depressed entitlement, attributing fewer resources to themselves, especially when average salaries by nationality are salient (see also Jost, 1997).

Thus far, however, our work shows that actual hierarchy and perceptions of that hierarchy are used as anchors to help make predictions about how individuals will be treated by institutions. The overall expectation is that wealth begets wealth: dominant groups are favored, and disadvantage is maintained. These findings replicate previous work in a new global context showing that stereotypes may help explain an existing social hierarchy, and that this may especially be the true for disadvantaged group members (Jost et al., 2003). This work also suggests a new way that local systems may be justified – by pointing to the global hierarchy. In showing a connection between local and global systems, we also demonstrate a new form of spreading rationalization, using the hierarchy from one system to explain (and perhaps defend) another.

## Conclusion

Although some consultants suggest that the current nationality-based economic hierarchy may disappear within the next decade (Anderson, 2014), perceptions of the current hierarchy in the UAE are strong and shared. Residents seem to understand this hierarchy as reflective of national differences in wealth and skill, with perceptions of national wealth providing a stronger explanation for individuals from disadvantaged regions. To the extent that such explanations are legitimized, these explanations may help maintain and reinforce both local and global inequality.

## Conflict of Interest Statement

The authors declare that the research was conducted in the absence of any commercial or financial relationships that could be construed as a potential conflict of interest.
